# Predictors of improvement in distance presenting visual acuity after refraction and reading rehabilitation at ≤ 1.5 M print size in older adults with low vision

**DOI:** 10.1371/journal.pone.0335003

**Published:** 2026-10-29

**Authors:** Daniel Martins Cabal, Manuela Molina Ferreira, Rosalia Antunes-Foschini

**Affiliations:** 1 Ribeirão Preto Medical School, University of São Paulo, Ribeirão Preto, Brazil; 2 Departament of Ophthalmology, Ribeirão Preto Medical School, University of São Paulo, São Paulo, Brazil; PLOS: Public Library of Science, UNITED KINGDOM OF GREAT BRITAIN AND NORTHERN IRELAND

## Abstract

**Background:**

This study aimed to identify factors associated with improvement in distance presenting visual acuity (dpVA) after refraction and with successful reading rehabilitation among patients aged 50 years or older with low vision.

**Methods:**

We conducted a retrospective observational study using medical records from a low-vision rehabilitation center in Ribeirão Preto, São Paulo, Brazil, between 2009 and 2019. Improvement after refraction was defined as a gain of at least two 0.2 logMAR after manifest refraction for distance correction. Successful reading rehabilitation using spectacles and/or magnifiers was defined as the ability to read texts of 1.5 M or smaller. Candidate predictors included age, sex, dpVA, distance best-corrected visual acuity, anatomical region responsible for low vision, educational level, spherical equivalent, and cylinder power. Logistic regression was performed, with statistical significance set at *p* < 0.05.

**Results:**

Overall, 558 patients were included, with a mean age of 67 years. Retinal involvement was the most frequent anatomical finding. Improvement in dpVA after refraction occurred in 20.3% of patients, and dpVA and spherical equivalent were significant predictors of this outcome. Among 346 literate patients, 252 (72.8%) were able to read 1.5 M texts after rehabilitation. Successful reading rehabilitation was associated with better distance best-corrected visual acuity, retinal involvement, higher educational level, and female sex, whereas older age and worse distance best-corrected visual acuity were associated with lower odds of success.

**Conclusions:**

Clinical, refractive, and demographic factors were associated with dpVA improvement after refraction and successful reading rehabilitation in older adults with low vision.

## Introduction

Visual impairment increases with age [1–4]. In developing countries, approximately 82% of blind people and 65% of people with mild to severe visual loss were 50 years or older [5]. In 2017, the International Agency for the Prevention of Blindness reported that in Brazil (Tropical Latin America group comprising Brazil and Paraguay) there are 719,140 people with blindness and 3,487,324 people with moderate to severe visual impairment. Visual impairment, particularly in its moderate, severe, or worse forms significantly impacts quality of life across various domains [6]. Globally, approximately 293 million people have moderate or severe visual impairment (distance presenting visual acuity (dpVA) of 20/60 or worse in the better-seeing eye, or visual field of less than 20º), and 43.3 million people worldwide are blind [1].

According to the 2019 update of ICD-10, moderate visual impairment is defined as dpVA in the better-seeing eye of less than 0.3 decimal (greater than 0.5 logMAR) and greater than or equal to 0.05 decimal (less than or equal to 1.3 logMAR), or a visual field of less than 20°. Under ICD-11, moderate visual impairment is defined as dpVA in the better-seeing eye of less than 0.5 decimal (greater than 0.3 logMAR) and greater than or equal to 0.05 decimal (less than or equal to 1.3 logMAR), or a visual field of less than 20°. Blindness is defined in both ICD-10 and ICD-11 as dpVA of less than 0.05 decimal (greater than 1.3 logMAR), or a visual field of less than 10° [7].

Rehabilitation services for individuals with low vision (LV) or blindness, which correspond to moderate and severe visual impairment and blindness under ICD-10, are essential, yet important barriers to access remain. Despite global initiatives, including in Brazil, access is still uneven, and the availability of specialized professionals is limited [8]. Overcoming these challenges is critical to reducing the adverse consequences of visual impairment. During the assessment of visual function, the ophthalmologist may identify opportunities to prescribe spectacles or optical aids that optimize residual vision, [9] thereby enhancing independence in daily activities and promoting reintegration into different social contexts [10,11].

Refraction for distance correction is a fundamental component of low-vision assessment, as it may improve residual visual function regardless of prior optical correction use. Fonda reported in 1987 and 1994 that distance VA improved after refraction in 15% of patients with LV and in 29% of those with diabetic retinopathy [12,13]. Sunness et al.[14] found an improvement of more than 0.2 logMAR in 11% of 739 low-vision patients with a median VA of 20/80^-2^; and Guo [15] reported an improvement of 27.9%, although patients with mild LV were also included. Despite these findings, evidence from developing countries remains limited, even though socioeconomic status and low educational level may affect rehabilitation outcomes [16].

Evidence on improvement in distance VA after refraction for distance correction remains scarce, especially following the advent of newer treatments for glaucoma and retinal diseases, including minimally invasive procedures and agents that inhibit abnormal neovascularization [17,18]. Furthermore, few studies have evaluated reading rehabilitation outcomes in low-vision populations overall. Most have focused on specific groups, such as patients with age-related macular degeneration, [19] and therefore may not represent reading ability outcomes in a general visual rehabilitation setting, in which LV may arise from diverse ophthalmologic and systemic disorders [20–22]. According to Stelmack et al., [23] many activities of daily living are closely linked to reading, including checking e-mails, comparing grocery prices, reading recipes, reading small or fine print, interpreting a thermometer, viewing a menu, and reading handwritten material. The U.S. ISBN Agency [24] further notes that, for most printed books, body text typically uses a font size between 10 and 12 points. According to the National Academies, [25] 1.0 M corresponds to approximately 8-point print with an x-height of about 1.45 mm, 1.25 M to approximately 10-point print, and 1.6 M to approximately 12.5-point print. In light of this information, the ability to read texts with a print size of 1.5 M or smaller appears to be a reasonable functional outcome for individuals with LV undergoing rehabilitation, as it may allow the reading of standard 12-point texts. Accordingly, this study aimed to determine: [1] the proportion of LV patients who improved their dpVA after manifest refraction and the predictors associated with this improvement; and [2] the proportion who, after reading rehabilitation, were able to read texts at or below 1.5 M, a threshold generally adequate for many reading-related activities of daily living and for standard 12-point book print, together with the predictors associated with this outcome.

## Materials and methods

This retrospective observational analytical study included patients aged 50 years or older who attended the Low-Vision service at the Rehabilitation Center (CER) of the Hospital das Clínicas, Ribeirão Preto Medical School, University of São Paulo (HCFMRP-USP), from 2009 to 2019. Data were accessed between December 6, 2021, and January 1, 2024. The study was approved by the HCFMRP Research Ethics Committee (CAAE 58577316.8.0000.5440; approval number 5,133,908) and adhered to the tenets of the Declaration of Helsinki. Written informed consent was waived due to the retrospective nature of the study. During the study period, eligibility for referral to the LV service required dpVA worse than 0.5 logMAR or visual field defects causing visual impairment.

In the Brazilian Public Health System, patients with visual complaints are initially evaluated by general practitioners and referred to ophthalmologic, tertiary, and rehabilitation services as needed. At our rehabilitation service, patients are managed by a multidisciplinary team comprising an ophthalmologist, occupational therapist, physical educator, orthoptist, pedagogue, social worker, and psychologist. When indicated, spectacles, optical aids, and walking sticks are prescribed. The rehabilitation protocol begins with a comprehensive ophthalmologic evaluation performed by a low-vision specialized ophthalmologist, with emphasis on the patients’ functional visual difficulties. The assessment includes measurement of distance and near presenting VA (npVA), manifest refraction for distance correction, distance best-corrected visual acuity (dBCVA), near addition in spectacles, trials with handheld or stand magnifiers, and near BCVA, using spectacles and/or magnifiers (nBCVA). Biomicroscopy and fundoscopy are also performed whenever feasible. During the initial visit, patients undergo preliminary testing to determine the optical aids most appropriate for reading, followed by training in the use of the optical devices over approximately two to three follow-up visits, after which the final prescription is provided.

Demographic data included age, sex, and educational level, categorized into three groups: illiterate or semi-illiterate, up to incomplete elementary school, and complete elementary school or higher. Educational level was self-reported either during the ophthalmologic examination or at hospital registration. Clinical data included dpVA, defined as distance VA measured without spectacles or with the patient’s current optical correction in both eyes; and dBCVA, obtained after refraction whenever feasible. In our LV service, dBCVA is routinely measured using charts in logMAR or decimal notation, and VA is defined as the smallest line on which at least half of the optotypes are correctly identified.

Decimal VA notation values were later converted to logMAR notation. Visual acuity recorded as hand movements was assigned a value of 1.9 logMAR. The anatomical site responsible for LV in the better-seeing eye was classified according to Gilbert [26] as whole globe, cornea, lens, uvea, retina, optic nerve, other causes, or normal-appearing globe. Refraction data included spherical equivalent, calculated as spherical power plus half of cylindrical power, and cylindrical power alone. Near VA (either npVA or nBCVA) was categorized as the ability or inability to read texts of 1.5 M or smaller. Reading ability was considered positive when there was no record of difficulty such as “reading with difficulty or very slowly,” “only recognizing numbers,” “spelling separate letters,” or “reading separate words but not phrases.” Standardized Portuguese texts [27–29] were previously printed in Times New Roman font, in sizes ranging from 10 to 24 points.

Printed 12-point texts were considered equivalent to 1.5 M. The MNREAD-P reading chart (Minnesota Low-Vision Reading Test, Portuguese version) [30] was also used during rehabilitation, although not to assess critical print size or reading speed. Patients who could read only 1.6 M sentences and not smaller print on the MNREAD-P chart were classified as unable to read texts of 1.5 M or smaller. Near reading distance was usually selected by the patient during rehabilitation as the distance providing the best image clarity with spectacles or near optical aids. Accordingly, patients able to read only 1.6 M or larger print were considered unable to read 1.5 M-sized texts. For logistic regression analyses, spherical equivalent and cylindrical power were entered as absolute values. Distance refractive correction prescription was also recorded as yes or no.

Eligibility criteria were dpVA in the better-seeing eye worse than 0.5 logMAR but better than light perception, a non-treatable cause of reduced VA, and a complete ophthalmologic examination performed by an ophthalmologist. Exclusion criteria were VA limited to light perception or no light perception, treatable causes of LV, and incomplete ophthalmologic evaluation or absence of examination by an ophthalmologist.

Two outcomes were analyzed: [1] improvement of at least 2 lines in the dpVA of the eye with the better dBCVA after manifest refraction for distance correction; and [2] the ability to read texts of 1.5 M or smaller. For the first outcome, dpVA was compared with dBCVA obtained after refraction for distance correction, and the predictors evaluated were age, sex, educational level (three levels), dpVA, spherical equivalent, cylindrical power, and anatomical region. Improvement in near VA was assessed by comparing near npVA, measured with the patient’s current near correction, with post-rehabilitation near VA, that is, nBCVA, achieved using the distance refraction combined with plus lenses and/or optical aids. For this outcome, the predictors evaluated were age, sex, educational level, dBCVA, spherical equivalent, cylindrical power, and anatomical region. Educational level was categorized into two levels, since illiterate and semi-illiterate patients were excluded from this analysis. Logistic regression was performed using Stata/IC 15.1 (College Station, TX), with *p* < 0.05 considered statistically significant. Data are presented as p-values, odds ratios (ORs), and 95% confidence intervals (95% CIs). Only variables with *p* < 0.20 in the univariable analysis were included in the multivariable model. Spearman’s correlation test was used to assess the correlation between dpVA or dBCVA and age.

For the statistical analyses, the eye with the best dBCVA in each patient and its corresponding refractive errors were selected. If dBCVA was the same bilaterally, the eye with better dpVA was selected. When both dpVA and dBCVA were identical bilaterally, refractive error from the right eye was used. Missing data were handled using available-case analysis (pairwise deletion), such that each analysis included all observations with non-missing data for the variables of interest. As a result, sample size varied across analyses, as indicated in the tables. To avoid multicollinearity, only one of the potentially collinear variables was included in each model.

## Results

Among 637 patients seen at CER, HCFMRP, USP, between 2009 and 2019, 46 were excluded because of incomplete data, and 33 because of blindness or light perception only. A total of 558 LV patients (87.6% of our total sample), aged 50–95 years (mean 67.7 ± 11.0 years), were included. There was no significant difference between sexes (chi-square test, *p* = 0.53). Refraction data were available for 546 of 558 patients (97.8%). Of these, 111 (20.3%) showed improvement in dpVA of at least 0.2 logMAR after manifest refraction. Mean ± SD logMAR dpVA was 1.22 ± 0.32 among those who improved by 0.2 logMAR or more and 1.14 ± 0.37 among those who did not meet this improvement criterion. Age was not correlated with either dpVA or dBCVA (Spearman test, *p* = 0.109 and *p* = 0.06, respectively). Age was also not associated with dpVA after stratification into the groups 50–59, 60–69, 70–79, and 80 years or older (Fisher’s exact test, *p* = 0.964). Educational level data were available for 483 of 558 patients (86.6%): 78 (16.1%) were classified as illiterate or semi-illiterate, 290 (60.0%) had incomplete elementary education, and 115 (23.8%) had complete elementary education or higher. Table 1 summarizes the demographic and clinical characteristics of the study sample.

**Table 1:** Demographic and clinical characteristics of patients included in the study (n = 558).

Age – years		67.7 ± 11.0
50-59		159/558 (28.5%)
60-69		150/558 (26.9%)
70-79		154/558 (27.6%)
80-95		95/558 (17.0%)
Female sex		288/558 (51.6%)
Educational level	Illiterate or semi-illiterate	78/483 (16.1%)
	Incomplete elementary school	290/483 (60.0%)
	Complete elementary school or more	115/483 (23.8%)
Most frequent anatomical region (retina)		401/557 (71.9%)
Presenting VA in the better eye (logMAR) (n = 556)		1.16 ± 0.36
Best-corrected visual acuity – better eye (logMAR) (n = 547)		1.09 ± 0.37
Cylinder – better eye (n = 483)		−1.25 ± 1.33
Spherical equivalent – better eye (n = 483)		−0.58 ± 3.78
Distance refractive correction prescription		168/557 (30.2%)
Prescription of optical devices		299/557 (53.7%)
dpVA improvement greater than or equal to 0.22 logMAR		111/546 (20.3%)
Reading ability at ≤1.5 M print size texts		/346 (73.1%)

The number of patients with available data for each variable is indicated in parentheses (n) Quantitative variables are presented as mean ± standard deviation, and categorical variables as percentages of the total.

Table 2 shows the results of the univariable and multivariable logistic regression analyses of improvement in dpVA after manifest refraction.

**Table 2:** Predictors of improvement in distance presenting visual acuity after manifest refraction.

	Univariable analysis			Multivariable analysis (n = 476)		
Variable	OR	95% IC	p	OR	95% IC	*p*
**dpVA**^**a**^ **(n=546)**	1.06	1.00–1.12	0.051	**1.13**	**1.06–1.21**	**<0.001**
**dpVA**^**b**^ **profound LV (n=122/546)**	**1.00**	**Reference**		---	---	---
**dpVA**^**b**^ **severe LV (n=140/546)**	**4.62**	**1.84–11.61**	**0.001**	---	---	---
**dpVA**^**b**^ **moderate LV (n=284/546)**	**7.32**	**3.10–17.32**	**<0.001**	---	---	---
Male sex (n = 546)	1.12	0.74–1.70	0.590	---	---	---
Age (n = 546)	1.00	0.98–1.02	0.953	---	---	---
EL (illiterate) (76/476)	1.00	Reference		---	---	---
EL (incomplete elementary) (286/476)	1.60	0.79 −3.22	0.186	---	---	---
EL (complete elementary or higher) (114/476)	1.49	0.68 −3.28	0.317	---	---	---
**Sph. Eq.**^**c**^ **(n = 478)**	**1.08**	**1.01 −1.15**	**0.018**	**1.07**	**1.00-1.14**	**0.035**
**Cylindrical power**^**c**^ **(n = 478)**	**1.17**	**1.00–1.36**	**0.045**	---	---	---
Anatomic region^d^ (n = 56/543)	1.00	Reference				
Anatomic region (cornea) (n = 11/543)	4.18	1.18-14.80	0.138	3.92	0.72-21.21	0.113

EL: Education Level; LV: low vision; Sph. Eq: Spherical equivalent.

^a^Increases of 0.1 logMAR units; ^b^dpVA: dpVA also evaluated based on the categories of visual impairment: profound (logMAR 1.4–1.9); severe (logMAR 1.1–1.3); moderate (logMAR ≤1.0); ^c^spherical equivalent and cylindrical power were analyzed as absolute values. Odds ratios are expressed per 1.0 D increase in the absolute value of each variable; ^d^Reference: glaucoma/the entire globe.

Regarding reading ability, data were available for 346 of the 405 patients (85.4%) considered eligible for reading rehabilitation assessment, namely those with incomplete elementary education, complete elementary education, or a higher educational level. Of these, 252 (72.8%) were able to read texts of 1.5 M or smaller, and 207 (59.8%) read texts sized less than or equal to 1.0 M. Among the 252 patients who were able to read 1.5 M text after rehabilitation, 69 (27.4%) had been able to read only 3–16 M text before rehabilitation, 98 (38.9%) had been able to read 1.6 to 2.5 M text, and 76 (30.2%) had already been able to read texts of 1.5 M or smaller but sought further improvement in visual functioning. Among patients who regained the ability to read texts of 1.5 M or smaller, the mean near addition prescribed in spectacles was 8.01 ± 3.8 D, with a range of 4–28 D. Prescribed powers for handheld and stand magnifiers ranged from 8 to 50 D, and the most frequently prescribed device was the 20 D handheld magnifier, followed by the 10 D and 50 D magnifiers. Patients were prescribed either spectacles, magnifiers, or a combination of both. Table 3 shows the predictors of reading rehabilitation. In the multivariable logistic regression model, dBCVA, age, sex, and retinal involvement jointly improved model fit compared with the null model, LR χ² [9] = 75.57, *p* < 0.001. Age not only remained independently associated with the outcome (OR = 0.95, 95% CI 0.92–0.97, *p* < 0.001), but also appeared to confound the association between dBCVA and reading rehabilitation, as adjustment for age meaningfully changed the effect estimate for dBCVA. Sex was not a confounder; it showed a weaker but statistically significant association with reading rehabilitation (OR = 0.54, 95% CI 0.31–0.94, *p* = 0.028). Although educational level was statistically significant in the univariable analysis (OR = 3.90, 95% CI 1.90–8.01, *p* < 0.001), this variable was not included in the multivariable model because data were available for only 297 individuals.

**Table 3:** Predictors of reading rehabilitation for texts less than or equal to 1.5 M in size.

	Univariable analysis			Multivariable analysis (n = 337)		
Variable	OR	IC 95%	p	OR	IC 95%	*p*
**dBCVA**^**a**^ **(n=345)**	**0.73**	**0.66–0.80**	**<0.001**	**0.69**	**0.62–0.77**	**<0.001**
**dBCVA**^**b**^ **profound LV (n=33)**	**1.0**	**Reference**		---	---	---
**dBCVA** ^**b**^ **severe LV (n=81)**	**3.91**	**1.64–9.32**	**0.002**	---	---	---
**dBCVA** ^**b**^ **moderate LV (n=231)**	**10.98**	**4.85–24.86**	**<0.001**	---	---	---
Male sex (n = 346)	0.67	0.41–1.08	0.098	**0.54**	**0.31–0.94**	**0.028**
**Age (n = 346)**	**0.96**	**0.94 - 0.98**	**0.001**	**0.95**	**0.92–0.97**	**<0.001**
**Educational level (CE) (n = 297)**	**3.90**	**1.90–8.01**	**<0.001**	---	---	---
Sph. Eq. (n = 330)	1.04	0.96–1.14	0.357	---	---	---
Cylindrical power (n = 330)	0.95	0.80–1.14	0.584	---	---	---
Anatomic region (all the globe) (23/338)	1.00	Reference				
Anatomic region (cornea)		empty				
Anatomic region (uvea) (12/338)	4.58	0.82–25.71	0.084	4.06	0.63–26.12	0.140
**Anatomic region (retina) (263/338)**	**2.63**	**0.82–25.71**	**0.028**	**3.24**	**1.18–8.87**	**0.022**
Anatomic region (normal ocular globe) (13/338)	3.06	0.66–14.08	0.152	1.16	0.214–6.37	0.858

CE: complete elementary school; Sph Eq.: Spherical Equivalent; LV: Low Vision

dBCVA^a^: increases of 0.1 logMAR.

dBCVA^b^: also evaluated based on the categories of visual impairment: profound (logMAR 1.4–1.9); severe (logMAR 1.1–1.3); moderate (logMAR ≤1.0).

## Discussion

Evidence on LV and rehabilitation remains limited worldwide, with the gap being even more pronounced in developing countries. Data from the Leiden study [3] involving individuals aged 85 years and older indicate that visual impairment predicts accelerated physical deterioration in later life. More recent findings further suggest that visual impairment is associated with a 34% higher risk of poor self-rated mental health and a 22% increase in symptoms of loneliness among older adults [31]. In addition, another study found a 47% higher risk of dementia in visually impaired older individuals [32]. In this context, a better understanding of the multiple dimensions of LV rehabilitation may help improve visual functioning, physical and cognitive outcomes, quality of life, and possibly survival [3].

Beyond their direct benefit to the population they serve, rehabilitation centers provide valuable data on comorbidities and on patients’ social and educational characteristics, which may inform future decision-making and improve planning of services for individuals with visual impairment. In Brazil, a country of continental dimensions, regional studies are especially important for clarifying the specific needs and demands of local populations [33–35].

In contrast to population-based studies showing an age-related decline in mean visual acuity, [36] we found no correlation between age and either dpVA or dBCVA.

In our sample, 111 patients (20.3%) experienced an improvement in dpVA of at least 0.2 logMAR. Interestingly, patients whose dpVA improved after refraction for distance correction had worse baseline dpVA, although this difference did not reach statistical significance. In a study of 707 older patients, [37] 38.8% had uncorrected refractive errors, and non-use of corrective lenses was even more common among those with ocular disease. In a case series of patients with LV of various etiologies and a mean logMAR VA of 0.92 ± 0.37, spectacle correction improved VA in 33.8% of patients [38]. Similarly, Sunness and Annan [14] reported an improvement of at least 0.2 logMAR after refraction in 11% of 739 patients with a VA of 20/80^-2^ (approximately 0.6 logMAR); and Guo et al.[15] reported an improvement of at least 0.2 logMAR in 27.9%; however, the study also included individuals with VA < 0.5 decimal (or >0.3 logMAR), meaning that patients with mild LV were included. These findings further support the importance of performing refraction in LV populations, particularly because improvement in visual function through optical correction may also alleviate depressive symptoms [39]. Evidence regarding quality of life, however, remains mixed. Although some studies involving patients with LV have shown positive effects, [40] a Cochrane review of adults with visual impairment, predominantly older individuals living in high-income countries, found only low- and moderate-certainty evidence of a small rehabilitation-related benefit in quality of life [5].

Although several studies, including systematic reviews, have shown that LV is more frequent in women than in men, [1] our study found no difference between sexes in the proportion of LV, nor any association between sex and improvement in dpVA after refraction. This discrepancy may be explained by the fact that, unlike earlier studies, ours was not population-based but rather reflects a cohort of patients attending a LV referral center. Likewise, educational level was not predictive of changes in dpVA after refraction in our sample. Therefore, even illiterate individuals with LV should be encouraged to undergo refraction for distance correction.

Among patients who improved dpVA after refraction, the median spherical equivalent was 1.87 D (interquartile range, 1.00 to 3.75 D), similar to values reported for the Brazilian population (hyperopia, 1.61 ± 1.27 D; myopia, 1.93 ± 2.38 D) [41]. In the univariable analysis, each 1.0D increase in the absolute value of the spherical equivalent (regardless the correction was hyperopic or myopic) was associated with an 8% increase in the odds of improvement in dpVA after refraction (OR = 1.08, 95% CI: 1.01–1.15) and by 7% in the multivariable analysis (OR = 1.07, 95% CI: 1.00–1.14). Guo et al.[15] reported similar findings, showing that each 1.0 D increase in spherical equivalent was associated with a 5% increase in the odds of improving VA by at least 0.2 logMAR (OR = 1.05, 95% CI: 1.01–1.09). Similarly, in the univariate analysis, each 1.0 D increase in the absolute value of cylindrical power was associated with a 17% increase in the odds of improving dpVA by at least 0.2 logMAR after refraction (OR = 1.17, 95% CI: 1.00–1.36). Although many patients with LV may not perceive substantial improvement in visual resolution with optical correction, the potential to reduce blur reinforces the importance of performing a refractive examination [42].

In our study, most patients with LV had retinal involvement (71.9%). Similarly, in a population-based study, Leasher et al.[43] identified macular degeneration and glaucoma as the leading causes of irreversible LV in South America. Data from the World Health Organization published in 2023 [44] also identify retinal diseases, including diabetic retinopathy and macular degeneration, as major causes of LV. We found no association between improvement in dpVA after refraction and the anatomical region affected. By contrast, a similar study including 2,923 participants found an association with corneal disease, with 34.8% of patients with corneal disorders showing VA improvement after refraction [15]. It is possible that our study lacked sufficient statistical power to detect a similar association with corneal disease.

After rehabilitation, 252 patients (72.8%) were able to read texts of 1.5 M or smaller. One study on reading rehabilitation reported an even higher proportion, with 94% of patients regaining the ability to read 10-point font text; however, their mean decimal VA was 0.18 ± 0.15, corresponding to approximately 0.74 logMAR, and all patients had age-related macular degeneration [19]. This is noteworthy, as retinal involvement also emerged as an independent predictor of successful reading rehabilitation in our multivariable analysis (OR = 3.24, 95% CI 1.18–8.87, *p* = 0.022). Another study found that only 2% of patients with LV could read a newspaper without optical aids, whereas approximately 51% were able to do so with optical aids [45].

Regarding the predictors for reading ability at 1.5 M, dBCVA and retinal involvement remained independently associated with reading rehabilitation after adjustment for age and sex. This finding is clinically plausible, as better baseline visual function is expected to favor reading performance after rehabilitation. Age appeared to confound the association between dBCVA and reading rehabilitation, suggesting that part of the crude association was influenced by the relationship between older age and poorer rehabilitation outcomes. However, age is also an independent predictor. In the multivariable analysis, each additional year of age was associated with a 5% reduction in the odds of being able to read texts of 1.5 M or smaller after rehabilitation (OR = 0.95, 95% CI 0.92–0.97). Previous studies have similarly shown that both reading ability and reading speed decline with age [46,47].

By contrast, sex had a smaller effect on the dBCVA estimate, suggesting a more limited confounding role. Even so, because both age and sex remained associated with the outcome in the adjusted model, their retention improved the interpretability of the final model.

The direct association between dBCVA and reading rehabilitation may also influence reading speed, [19,48] which is another determinant of reading rehabilitation. Indeed, other components of visual function, including contrast sensitivity, scotoma size, fixation stability and visual field (number of letters perceived simultaneously) are also known to influence rehabilitation outcomes [49]. However, assessment data for these parameters were not available in our study, because they are time-consuming and increase the number of hospital visits required, thereby delaying care for patients still awaiting evaluation in services already constrained by high demand and a limited number of LV specialists.

In our study, women showed a higher likelihood of successful reading rehabilitation than men, and sex appeared to be an independent predictor after adjustment for other covariates (Table 3). Evidence regarding sex-related differences in reading ability among individuals with LV is still limited. In a study examining this issue, [50] difficulty reading was the most frequently reported functional complaint (66.4%) regardless of sex. Sex-related differences were identified in other domains, with men more likely to report difficulties with driving and lighting, and women more likely to report difficulties with in-home activities, facial recognition, and social interaction. By contrast, a study involving individuals with normal vision and logMAR VA better than or equal to 0.1 [51] evaluated fifteen measures of visual perception, including VA, and found that men performed significantly better than women in six tests, whereas women did not perform better in any. This discrepancy may suggest that factors beyond basic visual performance have contributed to our findings. Cultural influences may be one possible explanation, since women often engage in activities that require good near vision, such as such as checking prices, expiration dates, and product ingredients while grocery shopping, or managing family members’ medications. Additionally, a survey found that although women make up 51% of Brazil’s population, they account for approximately 57% of book buyers [52].

Our study may have lacked sufficient power to detect associations between reading rehabilitation and certain anatomical regions, such as the cornea. In addition, neither spherical equivalent nor cylindrical power appeared to be associated with reading rehabilitation. However, we did not assess whether the type of astigmatism, namely with-the-rule or against-the-rule, could influence rehabilitation outcomes. Notably, one study suggested that with-the-rule astigmatism may have a greater negative effect on VA and near vision than against-the-rule astigmatism [53].

The present study identified retinal disease, particularly due to age-related macular degeneration, as the most common cause of LV in older adults. Among those who were able to read (n = 252), 195 (77.4%) had retinal involvement. These findings are consistent with a Cochrane systematic review, which identified retinal disease, particularly age-related macular degeneration, as the leading cause of LV in older adults [5]. The present study may have lacked sufficient statistical power to detect an association between reading rehabilitation outcomes and corneal involvement. Nevertheless, previous research has shown that the use of optical aids can significantly improve reading speed in patients with corneal disease [54].

This study has several limitations, primarily related to its retrospective nature. Educational background data were missing in 75 out of 558 (13.4%) of the medical records, and there was no information available on whether patients obtained the prescribed distance spectacles or consistently used optical aids for reading. Other determinants of reading rehabilitation, such as contrast sensitivity, scotoma size, fixation stability and visual field were not measured. Furthermore, the extended study period may have introduced temporal heterogeneity, including potential changes in referral patterns and patient characteristics over time. However, a key strength of this study is the standardized rehabilitation process, which was conducted by only two ophthalmologists and one orthoptist throughout the study period, and applied to a heterogeneous population with diverse etiologies of LV.

In conclusion, 20.3% of patients showed improvement in dpVA after manifest refraction for distance correction. Among literate patients, 73.1% were able to read texts of 1.5 M or smaller following reading rehabilitation. In multivariable analysis, baseline dpVA and spherical equivalent were independently associated with improvement of at least 0.2 logMAR in dpVA after refraction. Older age and worse dBCVA were associated with lower odds of successful reading rehabilitation, whereas retinal involvement and female sex were associated with higher odds of successful reading rehabilitation. Completion of elementary school education or higher was also associated with successful reading rehabilitation in the univariable analysis; however, educational level was not included in the multivariable model due to missing data.

## Supporting information

S1 Data Anonymized dataset PLOS ONE. (XLSX)
