# Supplementary material for: Predictors of improvement in distance presenting visual acuity after refraction and reading rehabilitation at ≤ 1.5 M print size in older adults with low vision
Source: PLoS One. 2026 Oct 29;21(10):e0335003. doi: 10.1371/journal.pone.0335003 (PMC13718947; doi:10.1371/journal.pone.0335003)
Supplement: Supplementary file 1 [file pone.0335003.s003.docx]

Ribeirão Preto, April 27, 2026.

Georgios Labiris, MD, PhD

Academic Editor

PLOS One

Dear Dr., Labiris,

Thank you for your comments and suggestions on our manuscript “Factors predicting Visual Acuity Improvement after Refraction and Reading Ability at 1.5 M in Patients with Low Vision”, PONE-D-25-53665.

We would also like to thank the Reviewers 1 and 2 for their suggestions and comments. The manuscript has been checked and revised as indicated.

Changes regarding the suggestions have been made in the new version of the manuscript (Revised Manuscript with track changes) and reviewed by all authors. Alterations made to the text have been highlighted using yellow mark. Detailed replies to the questions raised by the editor and the reviewers are given below.

We also take this opportunity to clarify that the funder (FAEPA) of this study had no role in the study design, data collection and analysis, decision to publish, or preparation of the manuscript. Below, the Editor and reviewers will find our point-by-point responses to the Editor’s comments.

We hope that the revised version of the manuscript will be suitable for publication in PLOS One as an Original Article. Thank you for your kind attention.

Sincerely Yours,

Rosalia Antunes-Foschini, MD, PhD

Associate Professor

Department of Ophthalmology, Otorhinolaryngology and Head&Neck Surgery, Ribeirão Preto Medical School, University of Sao Paulo

Av. Bandeirantes, 3900, CEP: 14049-900 – Ribeirão Preto SP, Brazil

antunesfoschini@gmail.com Phone/fax: 55-16-3602-2523

Reply for the Editor’s points:

**The manuscript addresses a clinically relevant topic with a valuable real-world dataset; however, several issues must be addressed prior to acceptance. The following changes are required:**

(i) **strengthen the clinical applicability of the findings by providing additional details, including thresholds of presenting visual acuity below which refraction is ineffective:**

**Answer: We added to the manuscript:**

“Mean ± SD logMAR VA was 1.22 ± 0.32 among those who improved by two or more lines and 1.14 ± 0.37 among those who did not improve., page 14.

**Answer: Regarding the mean near addition and lupes, added to the manuscript:**

“Among patients who regained the ability to read texts of 1.5 M or smaller, the mean near addition prescribed in spectacles was 8.01 ± 3.8 D, with a range of 4 to 28 D. Prescribed powers for handheld and stand magnifiers ranged from 8 to 50 D, and the most frequently prescribed device was the 20 D handheld magnifier, followed by the 10 D and 50 D magnifiers. Patients were prescribed either spectacles, magnifiers, or a combination of both.”, page 16

**and—if feasible—stratification by underlying ocular pathology;**

Answer: Tables 2 and 3 show the likelihoods of improvement in presenting visual acuity after manifest refraction and of successful reading near VA rehabilitation, defined as the ability to read texts of 1.5 M or smaller. In the univariable analysis, we further examined these outcomes according to the anatomical region involved. For the first outcome, (improvement in presenting visual acuity after manifest refraction), corneal involvement was the only anatomical variable with a p value < 0.20 and therefore entered the multivariable analysis. However, it was not statistically significant in the adjusted model. This issue is addressed in the Discussion section on page 20, at the end of the first paragraph. For the second outcome, retinal involvement emerged as a statistically significant anatomical predictor of successful reading rehabilitation, defined as the ability to read texts of 1.5 M or smaller; this finding is presented in Table 3 and discussed in pages 20 and 21, first and second paragraphs. Also, ans additional paragraph (page 21, first paragraph, highlighted was added, as follows:

…” Regarding the predictors for reading ability at 1.5 M, dBCVA and anatomic region retina remained the strongest independent predictors after adjustment for age and sex. This finding is clinically plausible, as better baseline visual function is expected to favor reading performance after rehabilitation. Age appeared to confound the association between dBCVA and reading rehabilitation, suggesting that part of the crude association was influenced by the relationship between older age and poorer rehabilitation outcomes. Age is also an independent predictor:”…

(ii) **expand the Methods to to better describe the rehabilitation process:**

Answer: We added the following text to explain the rehabilitation process:

“In the Brazilian Public Health System, patients with visual complaints are initially evaluated by general practitioners and referred to ophthalmologic, tertiary, and rehabilitation services as needed. At our rehabilitation service, patients are managed by a multidisciplinary team comprising an ophthalmologist, occupational therapist, physical educator, orthoptist, pedagogue, social worker, and psychologist. When indicated, spectacles, optical aids, and walking sticks are prescribed. The rehabilitation protocol begins with a comprehensive ophthalmologic evaluation performed by a low-vision specialized ophthalmologist, with emphasis on the patients’ functional visual difficulties. The assessment includes measurement of distance and near VA, manifest refraction, distance best-corrected visual acuity (dBCVA), near addition in spectacles, and trials with handheld or stand magnifiers. Biomicroscopy and fundoscopy are also performed whenever feasible. During the initial visit, patients undergo preliminary testing to determine the optical aids most appropriate for reading, followed by training in the use of the optical devices over approximately two to three follow-up visits, after which the final prescription is provided.” Page 11.

(iii) **Methods and Discussion: acknowledge key limitations, particularly the simplified definition of reading ability (≤1.5 M) and the absence of important visual function variables.**

Answer: We added a paragraph to the Introduction to clarify the rationale for selecting 1.5M as a threshold considered adequate for rehabilitation.

“. In light of this information, the ability to read texts with a print size of 1.5 M or smaller appears to be a reasonable functional outcome for individuals with LV undergoing rehabilitation, as it may allow the reading of standard 12-point texts.” Page 10

(iv) **(e.g., contrast sensitivity, visual fields, fixation stability);**

Answer: we added a paragraph to explain not performing some exams that are performed in prospective studies: …” however, our study does not have complete data of the assessments of all these parameters, because they are time-consuming and increase the number of hospital visits required, thereby delaying care for patients still awaiting evaluation in services already constrained by high demand and a limited number of low-vision specialists.” Pages 20-21

(v) **(iii) address potential heterogeneity due to the long study period and ensure more cautious interpretation of findings:**

Answer: We added the following paragraph to the discussion: “Other determinants of reading rehabilitation, such as contrast sensitivity, scotoma size, fixation stability and visual field were not measured. Furthermore, the extended study period may have introduced temporal heterogeneity, including potential changes in referral patterns, and patient characteristics over time.” Page 22

(vi) **including sex-related differences;**

Evidence on sex differences in low vision rehabilitation remains scarce, and published studies have generally not demonstrated significant differences between men and women. To address this point, and others related to the predictors of reading rehabilitation, we rearranged the following paragraphs of the discussion section (pages 21 and 22) See below:

…” Regarding the predictors for reading ability at 1.5 M, dBCVA and anatomic region retina remained the strongest independent predictors after adjustment for age and sex. This finding is clinically plausible, as better baseline visual function is expected to favor reading performance after rehabilitation. Age appeared to confound the association between dBCVA and reading rehabilitation, suggesting that part of the crude association was influenced by the relationship between older age and poorer rehabilitation outcomes. Age is also an independent predictor: we found that each additional year of age was associated with a 5% reduction in the likelihood of being able to read texts of 1.5 M or smaller in the multivariable model. Previous studies have similarly shown that both reading ability and reading speed decline with age.(46, 47)

By contrast, sex had a smaller effect on the dBCVA estimate, suggesting a more limited confounding role. Even so, because both age and sex remained associated with the outcome in the adjusted model, their retention improved the interpretability of the final model.

~~We observed a~~ The direct association between dBCVA and reading rehabilitation ~~such that better dBCVA was associated with a higher likelihood of being able to read texts of 1.5 M or smaller. Furthermore, dBCVA~~ may also influence reading speed, (19, 48) which is another determinant of reading rehabilitation. Indeed, other components of visual function, including contrast sensitivity, scotoma size, fixation stability and visual field (number of letters perceived simultaneously) are also known to influence rehabilitation outcomes. (49) however, our study does not have complete data of the assessments of all these parameters, because they are time-consuming and increase the number of hospital visits required, thereby delaying care for patients still awaiting evaluation in services already constrained by high demand and a limited number of low-vision specialists.

In our study, women showed a higher likelihood of successful reading rehabilitation than men, and sex appeared to be an independent predictor after adjustment for other covariates (Table 3). Evidence regarding sex-related differences in reading ability among individuals with LV is still limited. In a study examining this issue,(50) reading was the most reported functional complaint (66.4%) regardless of sex. Sex-related differences were identified in other domains, with men more likely to report difficulties with driving and lighting, and women more likely to report difficulties with in-home activities, facial recognition, and social interaction. By contrast, a study involving individuals with normal vision and logMAR VA ~~less~~ better than or equal to 0.1(51) evaluated fifteen measures of visual perception, including VA, and found that men performed significantly better than women in six tests, whereas women did not perform better in any. This discrepancy may suggest that factors beyond basic visual performance have contributed to our findings. Cultural influences may be one possible explanation, since women often engage in activities that require good near vision, such as grocery shopping - where they need to check prices, expiration dates, and product ingredients - or managing family members' medications. Additionally, a survey found that although women make up 51% of Brazil’s population, they account for approximately 57% of book buyers.(52)

We added a new reference: 50. Brown JC, Goldstein JE, Chan TL, Massof R, Ramulu P, Low Vision Research Network Study G. Characterizing functional complaints in patients seeking outpatient low-vision services in the United States. Ophthalmology. 2014;121(8):1655-62 e1.

(vii) **(iv) clarify statistical methods (handling of missing data):**

Answer: We added to the methods section the following explanation: “Missing data were handled using available-case analysis (pairwise deletion), such that each analysis included all observations with non-missing data for the variables of interest. As a result, sample size varied across analyses, as indicated in the tables.” Page 14

(viii) **assessment of multicollinearity:**

Answer: we added to methods section: “To avoid multicollinearity, only one of the potentially collinear variables was included in each model.” Page 14

(ix) **and (v) ensure consistency in visual acuity reporting**

Answer: We revised the manuscript throughout and retained both decimal and logMAR visual acuity values in the Introduction to facilitate comprehension for readers who are less familiar with the logMAR scale. We also retained decimal values when they were reported as such in the cited literature. These values are highlighted in the text to facilitate readability of the reviewers.

(x) **and correct language/typographical issues throughout.**

Answer: abstract: We improved English throughout the manuscript using AI (chat-GPT 5.4), keeping special attention so as not to change the meaning of the text. In addition, all newly added paragraphs are highlighted in yellow.

Below, the Editor and reviewers will find our point-by-point responses to the Reviewer 1’s comments.

**Reviewer #1: This study presents a valuable contribution by highlighting the role of refraction alone in low vision rehabilitation. The findings demonstrate that approximately 20% of patients experienced improvement in distance visual acuity, and 73% showed improvement in near vision, underscoring the clinical relevance of optimizing refractive correction before considering advanced low vision interventions.
However, while the stated aim of the study was to identify factors predicting visual acuity improvement after refraction and reading ability at 1.5 M in patients with low vision, the conclusions could be strengthened by providing additional clinically actionable details.**

**1) Specifically, it would be informative to report the lower cut off of presenting visual acuity (pVA) below which patients did not demonstrate improvement with refraction alone and therefore required a trial of low vision devices. Such information would assist clinicians in identifying patients who are unlikely to benefit from refraction-only strategies.**

Answer: We would like to thank Reviewer 1 for the thoughtful comments. The requested information has been incorporated into the manuscript, in page 14, as written below:

“Mean ± SD logMAR pVA was 1.22 ± 0.32 among those who improved by two or more lines and 1.14 ± 0.37 among those who did not improve., page 14.

**2) Regarding presenting visual acuity
…In addition, reporting the mean near addition prescribed would add significant value. Higher near additions (for example, +3.50 D) may reasonably be considered part of refractive management and have the potential to yield meaningful improvements in near visual acuity. This has important implications for primary eye care practice, as it suggests that basic refractive interventions could substantially improve reading ability without requiring immediate referral to a low vision rehabilitation specialist.**

Answer: Thank you for this important suggestion. We added information related to mean near addition and types of lupes to the results section (page 16) , as you can read below:

“Among patients who regained the ability to read texts of 1.5 M or smaller, the mean near addition prescribed in spectacles was 8.01 ± 3.8 D, with a range of 4 to 28 D. Prescribed powers for handheld and stand magnifiers ranged from 8 to 50 D, and the most frequently prescribed device was the 20 D handheld magnifier, followed by the 10 D and 50 D magnifiers. Patients were prescribed either spectacles, magnifiers, or a combination of both. Table 3 shows the predictors of reading rehabilitation. In the multivariable logistic regression model, dBCVA, age, sex, and anatomic region retina jointly improved model fit compared with the null model, LR χ²(9) = 75.57, p < 0.001. Age not only remained independently associated with the outcome (OR = 0.95, 95% CI 0.92–0.97, p < 0.001), but also appeared to confound the association between dBCVA and reading rehabilitation, as adjustment for age meaningfully changed the effect estimate for dBCVA. Sex was not a confounder; it showed a weaker but statistically significant association with reading rehabilitation (OR = 0.54, 95% CI 0.31–0.94, p = 0.028). Although educational level was statistically significant in the univariable analysis (OR = 3.90, 95% CI 1.90–8.01, p < 0.001), this variable was not included in the multivariable model because data were available for only 297 individuals.

”

**3) Furthermore, if the available records permit, stratifying outcomes by specific ocular pathologies—such as age-related macular degeneration (ARMD), diabetic retinopathy (DR), or glaucoma—would be useful. Identifying which underlying conditions are more likely to respond to refraction alone would enhance the clinical applicability of the findings and aid in patient counselling.**

Answer: Tables 2 and 3 show the likelihoods of improvement in presenting visual acuity after manifest refraction and of successful reading near VA rehabilitation, defined as the ability to read texts of 1.5 M or smaller. In the univariable analysis, we further examined these outcomes according to the anatomical region involved. For the first outcome, (improvement in presenting visual acuity after manifest refraction), corneal involvement was the only anatomical variable with a p value < 0.20 and therefore entered the multivariable analysis. However, it was not statistically significant in the adjusted model. This issue is addressed in the Discussion section on page 20, at the end of the first paragraph. For the second outcome, retinal involvement emerged as a statistically significant anatomical predictor of successful reading rehabilitation, defined as the ability to read texts of 1.5 M or smaller; this finding is presented in Table 3 and discussed in pages 20 and 21, first and second paragraphs. Also, ans additional paragraph (page 21, first paragraph, highlighted was added, as follows:

…” Regarding the predictors for reading ability at 1.5 M, dBCVA and anatomic region retina remained the strongest independent predictors after adjustment for age and sex. This finding is clinically plausible, as better baseline visual function is expected to favor reading performance after rehabilitation. Age appeared to confound the association between dBCVA and reading rehabilitation, suggesting that part of the crude association was influenced by the relationship between older age and poorer rehabilitation outcomes. Age is also an independent predictor:”…

**4) Finally, consistency in the reporting of visual acuity units should be ensured throughout the manuscript. For example, in lines 101–102, a value of 0.05 appears to be expressed in decimal notation, whereas LogMAR units are used elsewhere. It would be preferable to maintain a single visual acuity format throughout or clearly specify the units when different formats are used, in line with ICD 11 recommendations.**

Answer: We revised the manuscript throughout and retained both decimal and logMAR visual acuity values in the Introduction to facilitate comprehension for readers who are less familiar with the logMAR scale. We also retained decimal values when they were reported as such in the cited literature. These values are highlighted in the text to facilitate readability of the reviewers.

Below, the Editor and reviewers will find our point-by-point responses to the Reviewer’s 2 comments.

**Reviewer #2: Manuscript Title:
Factors predicting Visual Acuity Improvement after Refraction and Reading Ability at 1.5 M in Patients with Low Vision

General Comments

This manuscript presents a retrospective observational study investigating predictors of visual acuity improvement after refraction and predictors of reading rehabilitation in patients aged 50 years and older with low vision. The study analyzes medical records from 558 patients evaluated at a low-vision rehabilitation center in Ribeirão Preto, Brazil, between 2009 and 2019.

The topic is clinically relevant because refractive correction and visual rehabilitation remain fundamental interventions in low-vision care. Identifying factors associated with improved visual acuity and reading ability may help optimize patient selection and rehabilitation strategies in low-vision services.

Overall, the manuscript addresses an important clinical question and includes a relatively large sample size for a single-center low-vision cohort. The use of logistic regression to identify predictors is appropriate. However, several methodological and reporting issues should be addressed before the manuscript can be considered for publication.**

**Major Comments
1. Definition of Reading Rehabilitation Outcome

The study defines successful reading rehabilitation as the ability to read texts sized ≤1.5 M. While this threshold may be clinically meaningful, reading ability in low-vision research is typically assessed using additional parameters such as: reading speed, critical print size, maximum reading speed. Although the MNREAD chart is mentioned, it was not used to measure these parameters. This limitation should be more clearly discussed, as reading ability is multidimensional and cannot be fully captured by a single print-size threshold.**

Answer: We agree with Reviewer 2 on how important and meaningful the assessment of other parameters are for assessing reading rehabilitation. However, we do not perform all these tests routinely, due to be time-consuming, delaying the assistance of other patients with LV who need rehabilitation. We added some paragraphs to the introduction, methods and discussion to address this subject:

(i) **Methods and Discussion: acknowledge key limitations, particularly the simplified definition of reading ability (≤1.5 M) and the absence of important visual function variables.**

Answer: We added a paragraph to the Introduction to clarify the rationale for selecting 1.5M as a threshold considered adequate for rehabilitation, a rationale to justify the 1.5 M size:

“. In light of this information, the ability to read texts with a print size of 1.5 M or smaller appears to be a reasonable functional outcome for individuals with LV undergoing rehabilitation, as it may allow the reading of standard 12-point texts.” Page 10

(ii) **(e.g., contrast sensitivity, visual fields, fixation stability);**

Answer: we added a paragraph to explain not performing some exams that are performed in prospective studies: …” however, our study does not have complete data of the assessments of all these parameters, because they are time-consuming and increase the number of hospital visits required, thereby delaying care for patients still awaiting evaluation in services already constrained by high demand and a limited number of low-vision specialists.” Pages 20-21

(iii) **(iii) address potential heterogeneity due to the long study period and ensure more cautious interpretation of findings:**

Answer: We added the following paragraph to the discussion: “Other determinants of reading rehabilitation, such as contrast sensitivity, scotoma size, fixation stability and visual field were not measured. Furthermore, the extended study period may have introduced temporal heterogeneity, including potential changes in referral patterns, and patient characteristics over time.” Page 22

**2. Lack of Important Visual Function Variables

Several key factors known to influence reading performance in low vision were not evaluated, including: contrast sensitivity, central scotoma characteristics, visual field parameters, fixation stability
These variables are important determinants of reading ability and should be acknowledged more explicitly as limitations in the Discussion section.**

Answer: We added to the paragraph of limitations (page 22) the following paragraph:

…” Other determinants of reading rehabilitation, such as contrast sensitivity, scotoma size, fixation stability and visual field were not measured.

**3. Long Study Period and Potential Heterogeneity

The study includes patients from a ten-year period (2009–2019). During this time, substantial advances occurred in the treatment of retinal diseases and glaucoma, including anti-VEGF therapy and minimally invasive glaucoma procedures.
These developments may have influenced the characteristics of patients referred for low-vision rehabilitation. The authors should comment on the potential impact of this long inclusion period on the study findings.**

Answer: we added a paragraph (page 24) commenting potential changes in referral patterns over time, as follows:

Furthermore, the extended study period may have introduced temporal heterogeneity, including potential changes in referral patterns, and patient characteristics over time.”


**4. Interpretation of Sex Differences

The study reports that female sex was associated with a higher likelihood of successful reading rehabilitation. The explanation proposed in the Discussion suggests cultural or behavioral factors such as reading habits and daily activities.

This interpretation appears somewhat speculative and should therefore be presented more cautiously. Alternative explanations or the possibility of residual confounding should be discussed.**

(i) **including sex-related differences;**

Answer: We rearranged some paragraphs of the discussion to better discuss the predictors of reading rehabilitation, as follows:

“…Regarding the predictors for reading ability at 1.5 M, dBCVA and anatomic region retina remained the strongest independent predictors after adjustment for age and sex. This finding is clinically plausible, as better baseline visual function is expected to favor reading performance after rehabilitation. Age appeared to confound the association between dBCVA and reading rehabilitation, suggesting that part of the crude association was influenced by the relationship between older age and poorer rehabilitation outcomes. Age is also an independent predictor: we found that each additional year of age was associated with a 4% reduction in the likelihood of being able to read texts of 1.5 M or smaller. Previous studies have similarly shown that both reading ability and reading speed decline with age.(46, 47)

By contrast, sex had a smaller effect on the dBCVA estimate, suggesting a more limited confounding role. Even so, because both age and sex remained associated with the outcome in the adjusted model, their retention improved the interpretability of the final model.

~~We observed a~~ The direct association between dBCVA and reading rehabilitation ~~such that better dBCVA was associated with a higher likelihood of being able to read texts of 1.5 M or smaller. Furthermore, dBCVA~~ may also influence reading speed, (19, 48) which is another determinant of reading rehabilitation. Indeed, other components of visual function, including contrast sensitivity, scotoma size, fixation stability and visual field (number of letters perceived simultaneously) are also known to influence rehabilitation outcomes. (49) however, our study does not have complete data of the assessments of all these parameters, because they are time-consuming and increase the number of hospital visits required, thereby delaying care for patients still awaiting evaluation in services already constrained by high demand and a limited number of LV specialists.

In our study, women showed a higher likelihood of successful reading rehabilitation than men, and sex appeared to be an independent predictor after adjustment for other covariates (Table 3). Evidence regarding sex-related differences in reading ability among individuals with LV is still limited. In a study examining this issue,(50) reading was the most reported functional complaint (66.4%) regardless of sex. Sex-related differences were identified in other domains, with men more likely to report difficulties with driving and lighting, and women more likely to report difficulties with in-home activities, facial recognition, and social interaction. By contrast, a study involving individuals with normal vision and logMAR VA ~~less~~ better than or equal to 0.1(51) evaluated fifteen measures of visual perception, including VA, and found that men performed significantly better than women in six tests, whereas women did not perform better in any. This discrepancy may suggest that factors beyond basic visual performance have contributed to our findings. Cultural influences may be one possible explanation, since women often engage in activities that require good near vision, such as grocery shopping - where they need to check prices, expiration dates, and product ingredients - or managing family members' medications.

1) Final mutivariable logistic regression

As the changes in the OR of dBCVA were approximately 8% (0.039 and 0.042) , and based on the pratical rule of 10% change in the OR, sex is not considered an important confounder here, or it is a minor confounder.

We added a paragraph to the results section with this explanation, as shown below (page 17)

…” . In the multivariable logistic regression model, dBCVA, age, sex, and anatomic region retina jointly improved model fit compared with the null model, LR χ²(9) = 75.57, p < 0.001. Age not only remained independently associated with the outcome (OR = 0.95, 95% CI 0.92–0.97, p < 0.001), but also appeared to confound the association between dBCVA and reading rehabilitation, as adjustment for age meaningfully changed the effect estimate for dBCVA. Sex was not a confounder; it showed a weaker but statistically significant association with reading rehabilitation (OR = 0.54, 95% CI 0.31–0.94, p = 0.028). Although educational level was statistically significant in the univariate analysis (OR = 3.90, 95% CI 1.90–8.01, p < 0.001), this variable was not included in the multivariable model because data were available for only 297 individuals.”…

**5. Description of the Rehabilitation Process

The manuscript would benefit from a more detailed description of the rehabilitation protocol, including: types of optical aids prescribed, number of rehabilitation sessions, duration of training, criteria for selecting visual aids
Answer:

This information is important for reproducibility and for readers to interpret the results in the context of clinical practice.

Minor Comments
1. Language and grammar

The manuscript is generally understandable, but several sentences would benefit from editing by a native English speaker to improve clarity and readability.

Examples include minor grammatical inconsistencies and wording issues throughout the manuscript.**

Answer: We revised the entire manuscript and improved English throughout the manuscript using AI (chat-GPT 5.4), keeping special attention so as not to change the meaning of the text.

**2. Typographical errors:

There are occasional typographical errors, for example:
“manisfest refraction” in Table 2; inconsistent spacing in some sections

Thank you for the review. We revised all the manuscript, looking for typographical errors, inconsistent spacing, and abbreviations.
3. Clarification of statistical reporting

The manuscript reports odds ratios and confidence intervals appropriately. However, the Methods section should clarify:

how missing data were handled; whether multicollinearity between predictors was assessed**

Answer: This information was added to the methods section, as shown below:

“Missing data were handled using available-case analysis (pairwise deletion), such that each analysis included all observations with non-missing data for the variables of interest. As a result, sample size varied across analyses, as indicated in the tables” “To avoid multicollinearity, only one of the potentially collinear variables was included in each model.” Page 14

**4. Title clarity

The title is generally appropriate, but the phrase “Reading Ability at 1.5 M” could be clarified as: “Reading Ability at ≤1.5 M Print Size”**

Answer: We agree with the Reviewer and changed the title to:

Factors predicting Visual Acuity Improvement after Refraction and Reading Ability at ≤1.5 M Print Size in Patients with Low Vision
