# Supplementary material for: Predictors of improvement in distance presenting visual acuity after refraction and reading rehabilitation at ≤ 1.5 M print size in older adults with low vision
Source: PLoS One. 2026 Oct 29;21(10):e0335003. doi: 10.1371/journal.pone.0335003 (PMC13718947; doi:10.1371/journal.pone.0335003)
Supplement: Supplementary file 2 [file pone.0335003.s004.docx]

Ribeirão Preto, June 16^th^, 2026.

Georgios Labiris, MD, PhD

Academic Editor

PLOS One

Dear Dr., Labiris,

Thank you for your comments and suggestions on our manuscript “Factors predicting Visual Acuity Improvement after Refraction and Reading Ability at 1.5 M in Patients with Low Vision”, PONE-D-25-53665.

We would also like to thank the Reviewer 1 for the suggestions. The manuscript has been checked and revised as indicated.

Changes regarding the suggestions have been made in the new version of the manuscript and reviewed by all authors. Alterations made to the text have been highlighted using yellow mark. Replies to the concerns raised by reviewer 1 are given in the reply cover letter-review2.

Sincerely Yours,

Rosalia Antunes-Foschini, MD, PhD

Associate Professor

Department of Ophthalmology, Otorhinolaryngology and Head&Neck Surgery, Ribeirão Preto Medical School, University of Sao Paulo

Av. Bandeirantes, 3900, CEP: 14049-900 – Ribeirão Preto SP, Brazil

antunesfoschini@gmail.com Phone/fax: 55-16-3602-2523

**Editor: Reviewer 1 raises one remaining issue regarding the use of the term “refraction,” as the study includes both spectacle correction and low vision devices. Before the manuscript can be accepted, please revise the terminology throughout the manuscript (including the title) to accurately reflect the interventions assessed (e.g., “best corrected near visual acuity”), or alternatively restrict the analysis to spectacle correction alone. This is the only required revision. Subject to satisfactory resolution of this point, the manuscript meets PLOS ONE’s publication criteria.**

**Reviewer #1: My only concern is when the aim of the study reads improvement with refraction, Ideally it would mean only spectacle correction, it could be extended to spectacle magnifiers upto 8.00Ds. However the authors have also included hand and stand magnfiers that truly do not qualify as refraction but as low vision devices for near correction. Rest of the comments have been addressed well. They either remove the word refraction in the tiltle and manuscript and replace with best corrected near acuity, which actually beats the primary objective or include only those with spectacle correction.**

**Answer:** Thank you for this important observation. We understand your concern and agree that hand and stand magnifiers should not be classified as part of “refraction”, but rather as low-vision devices. Accordingly, we revised the title to clarify that the manuscript evaluates two distinct outcomes: improvement in distance presenting visual acuity (dpVA) after refraction for distance correction, and improvement in near presenting visual acuity (npVA) after the adaptation of spectacles, with additions up to +3.5 D, and/or low-vision devices, including additions of +4.0 D or higher, hand magnifiers, and stand magnifiers. We used the term near best-corrected visual acuity (nBCVA) to refer to visual acuity after reading rehabilitation with spectacle additions and/or magnifiers.

Changes in the manuscript are marked in yellow:

1)Title:

**Predictors of Improvement in Distance Presenting Visual Acuity after Refraction and Reading Rehabilitation at ≤1.5 M Print Size in Patients with Low Vision**

2) Troughout the manuscript:

dpVA (distance presenting visual acuity) in place of pVA (presenting visual acuity). All marked in yellow.

3) Troughout the manuscript, we wrote “refraction for distance correction” in place of “refraction”. All marked in yellow.

4) We added nBCVA (near best-corrected visual acuity) to refer to the near acuity after reading rehabiliatation. All marked in yellow.

5) These terms were changed also on the tables, and are marked in yellow.
